# Fecal microbiota transplantation for patients with irritable bowel syndrome: A meta-analysis protocol

**DOI:** 10.1097/MD.0000000000012661

**Published:** 2018-10-05

**Authors:** Wenting Wen, Haibo Zhang, Junlong Shen, Luxia Wei, Shunong Shen

**Affiliations:** Nanjing University of Chinese Medicine, Nanjing, China.

**Keywords:** fecal microbiota transplantation, irritable bowel syndrome, meta-analysis, protocol

## Abstract

Irritable bowel syndrome (IBS) is a common functional bowel disease characterized by chronic or recurrent abdominal pain, bloating, constipation, and diarrhea. Many patients with IBS have a poor quality of life due to abdominal discomfort, diarrhea, constipation, and the presence of other diseases. At present, intestinal motility inhibitors, adsorbents, astringents, intestinal mucosal protective agents, and antidepressants have been combined to treat IBS, but the treatment process is long, which results in a large economic burden to patients. Fecal microbiota transplantation (FMT) is a treatment involving the transplantation of functional bacteria from healthy human feces into the gastrointestinal tract of patients; thus, replacing the intestinal flora and modulating intestinal and extra-intestinal diseases. In recent years, the efficacy and economic benefits of FMT in the treatment of IBS have received increasing attention from researchers.

A search for randomized controlled trials (RCTs) on treating IBS with FMT will be performed using 9 databases, including PubMed, the Cochrane Library, Embase, ClinicalTrails, China National Knowledge Infrastructure, Sino Med, ScienceDirect, VIP, and Wanfang Data. Two reviewers will independently screen data extraction studies and assess study quality and risk of bias. The risk of bias for each RCT will be assessed against the Cochrane Handbook standards to assess methodological quality. RevMan V.5.3 software will be used to calculate data synthesis when meta-analysis is allowed.

This study will provide a high-quality synthesis of existing evidence on the effectiveness and safety of FMT in the treatment of IBS.

This study will determine if FMT is an effective and safe intervention for IBS.

PROSPERO registration number is PROSPERO CRD42018108080.

## 1 Introduction

Irritable bowel syndrome (IBS) is a chronic, recurrent functional gastrointestinal disorder,^[1]^ characterized by changes in bowel habits or stool features, continuous or intermittent abdominal pain, and bloating or abdominal discomfort,^[2]^ with a prevalence of up to 10% to 20% in the United Kingdom and the United States^[3,4]^ and a prevalence of 5% to 10% in most Asian countries.^[5,6]^ Recent studies show that the prevalence of IBS in Asian and African regions increases as the economy grows.^[3,4]^ There are a number of factors to be involved in the pathogenesis of IBS, including impaired motility and sensitivity, increased permeability, changes in the gut microbiome, and alterations in the brain–gut axis.^[7,8]^ The first presentation of IBS patient to a physician is mostly between the age of 30 and 50 years, and the symptoms’ onset is closely related to psychological stress.^[3]^ Individuals with IBS have a 2-fold higher risk of comorbid depression, anxiety, and sleep disorders. Conversely, 25% to 30% of patients with depression and 10% to 45% of patients with anxiety are reported to develop IBS.^[9–11]^ The co-existence of chronic disease with psychiatric disorder can be a big burden, and comorbid depression has a stronger negative effect on health than depression or anxiety does alone.^[12]^ As measured by validated survey instruments, such as the Short Form-36, IBS has a negative impact on an affected patient's quality of life.^[13]^ Annually, IBS costs the US health system in excess of $30 billion.^[14]^ In recent years, combined administration of intestinal peristalsis inhibitors, adsorbents, astringents, intestinal mucosa protectors, and antidepressants in the treatment of IBS have achieved a certain therapeutic effect, but with a long therapeutic course and heavy economic burden for the patient, and with a high recurrence rate after ceasing the drugs.^[15]^ Therefore, the most urgent and intractable problem for clinical gastroenterologists to deal with is to develop a both effective and economical treatment method that could cope with different symptoms of IBS.^[16]^

Fecal microbiota transplantation (FMT), also known as a stool transplant, is the transplantation of fecal bacteria from a healthy individual into a recipient, often with the intent to cure intestine-associated diseases by rebuilding the intestinal microbial community structure.^[17]^ In ancient China, feces were commonly used to treat diseases. Published clinical trials and systematic evaluations support a positive role for FMT in treating inflammatory bowel disease.^[18]^ In recent years, researchers have begun to look to treating IBS with FMT, with an increasing number of trials having been published on the subject. The purpose of this meta-analysis is to systematically evaluate the efficacy and safety of FMT in the treatment of IBS and to provide recommendations for experimental research on clinical application of FMT in IBS.

## 2 Methods

### 2.1 Inclusion criteria for study selection

#### 2.1.1 Types of studies

All randomized controlled trials (RCTs) will be included without language limitations.

#### 2.1.2 Types of participants

Standard diagnosis of IBS patients will be included in the analysis, regardless of their age, gender, ethnicity, and background, according to the Manning and Rome I, II, III, and IV criteria.

#### 2.1.3 Types of interventions

In this study, the intervention received by patients with IBS in the experimental group will be fecal bacteria transplantation, while the control group will be administered a placebo.

#### 2.1.4 Result measurements

The main outcome assessed will be change in the symptom scores of patients with IBS after treatment compared to that before treatment, while the secondary outcomes measured will be changes in anxiety and depression symptoms and quality of life.

### 2.2 Retrieval strategy

#### 2.2.1 Electronic searches

To identify relevant information, the PubMed, Cochrane Library, Embase, ClinicalTrails, China National Knowledge Infrastructure, Sino Med, ScienceDirect, VIP, and Wanfang Data databases will be electronically searched. All the databases will be searched from their inception to March 1, 2019. No language restrictions will be applied to the search. Studies that do not meet the inclusion criteria will be excluded. Two reviewers (WW and HZ) will independently search the studies. Any differences will be resolved through discussion with a third author (JS).

Search strategy of PubMed was as follows:

(1) (((((FMT) or intestinal microbiota transfer) or fecal transplantation) or fecal transplant) or donor feces infusion) or FMT,
(2) ((((IBSs) or irritable colon) or mucous colitis) or mucous colitis) or IBS, and
(3) Step 1 and step 2.

#### 2.2.2 Searching other resources

Potential eligible studies will be searched for relevant conference proceedings and reference lists of previously published reviews.

### 2.3 Data collection and analysis

#### 2.3.1 Selection of studies

Two reviewers (WW and HZ) will independently search and evaluate every relevant study according to the Cochrane Handbook for Systematic Reviews of Interventions and perform the following:

1. eliminate irrelevant literature based on titles and abstracts,
2. organize the article results using NoteExpress software and eliminate any duplicates,
3. eliminate nonconforming literature based on the inclusion criteria by perusing the full text, and
4. amalgamating all literature that reports on identical clinical studies.

The selection process will be summarized according to the PRISMA flow diagram. The 2 reviewers will review the full text of these studies according to the standardized eligibility criteria table to assess their qualifications. Detailed reasons for cancellation of the trial will be recorded when screening papers. Any differences will be resolved through discussions between the 2 reviewers. If no consensus is reached during the discussion, an independent reviewer will be consulted.

#### 2.3.2 Data extraction and management

Data from each selected article will be extracted independently by WW and HZ. The general research information will include patient characteristics (gender, mean age, and mean duration of disease), diagnostic criteria (Rome I, II, III, or IV or Manning), interventions received by the experimental and control groups, and major and minor events. A third researcher, JS, will re-examine the data. If necessary, we will consult the author of the article for more information. Any possible differences will be resolved by arbitrators and experts.

#### 2.3.3 Processing missing data

When experimental data are missing or inadequate, we will attempt to contact the original author of the study by e-mail or telephone to obtain sufficient and comprehensive data. Incomplete data will be discarded if sufficient data cannot be retrieved.

#### 2.3.4 Assessment of risk of bias in included studies

Two reviewers (WW and HZ) will independently evaluate the risk of bias in the eligible studies using the Cochrane Collaboration's tool. Each study will be evaluated for validity based on the following 7 aspects: random sequence generation, allocation concealment, blinding of participants and personnel, blinding of outcome assessment, incomplete data assessment, selective outcome reporting, and other sources of bias, according to the Cochrane risk of bias standards.

#### 2.3.5 Heterogeneity assessment and statistical analysis

Review Manager 5.3 software will be utilized for statistical analysis on the basis of homogeneity of the included trials. Odds ratios and 95% confidence intervals (95% CIs) will be applied for dichotomous data; continuous data, weighted mean differences, and 95% CIs for analysis when units are the same, while standardized mean differences and 95% CIs will be used for analysis when units are different. The *I*^2^ statistic will be utilized for assessing the heterogeneity of the included trials. A fixed effects model will be applied to calculate the pooled statistics in the absence of substantial heterogeneity (*I*^2^ < 50% and *P* ≥ .1). Conversely, if statistical heterogeneity is identified (*I*^2^ > 50% or *P* < .1), the causes of the heterogeneity will be identified first by subgroup analysis. If the heterogeneity cannot be readily explained, a random effects model will be interpreted with caution.

#### 2.3.6 Subgroup analysis

If significant heterogeneity is observed in the included studies, subgroup analysis will be performed based on age, gender, interventions, controls, and outcome measurements.

#### 2.3.7 Assessment of reporting biases

If there are a sufficient number of articles (>10) included under the same endpoint addressing the same question, a funnel plot will be used to measure publication bias.

#### 2.3.8 Sensitivity analysis

If sufficient test data are available, sensitivity analysis will be performed to determine whether the conclusion is robust.

#### 2.3.9 Grading of quality of evidence

The Grading of Recommendations Assessment, Development, and Evaluation will be utilized for assessing the quality of evidence for the main outcomes. The quality of evidence will be categorized as high, moderate, low, or very low.

#### 2.3.10 Ethics and dissemination

Ethics approval is not required because the data will not include individual patient data and, therefore, will not incur raise any privacy issues. The results of this systematic review will be disseminated only in a peer-reviewed publication.

## 3 Discussion

IBS is a functional disorder of the gastrointestinal tract and its physiology is not very well-understood. Published studies confirm an association between IBS and disorders of the intestinal flora.^[19]^ The ideal composition of a healthy microbiome has not yet been defined,^[20]^ but the composition of the fecal microbiota in patients with IBS differs significantly from that in healthy controls, with several differences in bacterial species having been identified.^[21–35]^ Studies have shown that probiotic supplementation aids in the treatment of IBS, but high-quality studies tend to indicate a less notable therapeutic effect, and the compositions of probiotic supplements vary greatly.^[36]^ In recent years, FMT has attracted considerable attention for treatment of gastrointestinal microbe-related diseases. FMT has great potential to become a highly effective, low-cost treatment for IBS. The intestinal flora in fecal microbiota originates from healthy people, ensuring the diversity of the probiotics. An increasing number of RCTs have been published in clinical studies and, therefore, we will conduct this systematic review and meta-analysis to assess the efficacy and safety of FMT in the treatment of IBS.

To the best of our knowledge, this is the first analysis of the literature on the use of FMT to treat IBS. We hope this systematic review will provide strong evidence for the efficacy and safety of FMT in treating IBS, to be used by clinicians, researchers, and health-policy advisors.

## Author contributions

**Conceptualization:** Wenting Wen, Haibo Zhang, Junlong Shen and Shunong Shen

**Data curation:** Wenting Wen and Haibo Zhang

**Formal analysis:** Wenting Wen, Haibo Zhang, and Junlong Shen

**Writing:** Wenting Wen, Haibo Zhang, and Luxia Wei

**Funding acquisition:** Junlong Shen, Luxia Wei, and Wenting Wen

Wenting Wen orcid: 0000-0002-3930-818X
